# Dosimetric feasibility analysis and presentation of an isotoxic dose-escalated radiation therapy concept for glioblastoma used in the PRIDE trial (NOA-28; ARO-2022-12)

**DOI:** 10.1016/j.ctro.2023.100706

**Published:** 2023-12-03

**Authors:** Raphael Bodensohn, Daniel F. Fleischmann, Sebastian H. Maier, Vasiliki Anagnostatou, Sylvia Garny, Alexander Nitschmann, Marcel Büttner, Johannes Mücke, Stephan Schönecker, Kristian Unger, Elgin Hoffmann, Frank Paulsen, Daniela Thorwarth, Adrien Holzgreve, Nathalie L. Albert, Stefanie Corradini, Ghazaleh Tabatabai, Claus Belka, Maximilian Niyazi

**Affiliations:** a Department of Radiation Oncology, University Hospital Tübingen, Tübingen, Germany; b Center for Neuro-Oncology, Comprehensive Cancer Center Tübingen-Stuttgart, University Hospital Tübingen, Tübingen, Germany; c Department of Radiation Oncology, LMU University Hospital, LMU Munich, Munich, Germany; d German Cancer Consortium (DKTK), partner site Munich, a partnership between DKFZ and LMU University Hospital, Munich, Germany; e German Cancer Research Center (DKFZ), Heidelberg, Germany; f Bavarian Cancer Research Center (BZKF), Munich, Germany; g Helmholtz Zentrum Munich, Neuherberg, Germany; h Faculty of Medicine, LMU Munich, Munich Germany; i Section for Biomedical Physics, Department of Radiation Oncology, University Hospital Tübingen, Germany; j Department of Nuclear Medicine, LMU University Hospital, LMU Munich, Munich, Germany; k Department of Neurology and Interdisciplinary Neuro-Oncology, University Hospital Tübingen, Hertie Institute for Clinical Brain Research, Tübingen, Germany; l German Cancer Consortium (DKTK), partner site Tübingen, a partnership between DKFZ and University Hospital, Tübingen, Germany

**Keywords:** Glioblastoma, Dose escalation, FET PET, Bevacizumab, Radiation necrosis, NTCP

## Abstract

**Background and purpose:**

The PRIDE trial (NOA-28; ARO-2022-12; NCT05871021) is scheduled to start recruitment in October 2023. Its primary objective is to enhance median overall survival (OS), compared to historical median OS rates, in patients with methylguanine methlyltransferase (MGMT) promotor unmethylated glioblastoma by incorporating isotoxic dose escalation to 75 Gy in 30 fractions. To achieve isotoxicity and counteract the elevated risk of radiation necrosis (RN) associated with dose-escalated regimens, the addition of protective concurrent bevacizumab (BEV) serves as an innovative approach. The current study aims to assess the dosimetric feasibility of the proposed concept.

**Materials and methods:**

A total of ten patients diagnosed with glioblastoma were included in this dosimetric analysis. Delineation of target volumes for the reference plans adhered to the ESTRO-EANO 2023 guideline. The experimental plans included an additional volume for the integrated boost. Additionally, the 60 Gy-volume was reduced by using a margin of 1.0 cm instead of 1.5 cm. To assess the risk of symptomatic RN, the Normal Tissue Complication Probability (NTCP) was calculated and compared between the reference and experimental plans.

**Results:**

Median NTCP of the reference plan (NTCP_ref_) and of the experimental plan (NTCP_ex_) were 0.24 (range 0.11–0.29) and 0.42 (range 0.18–0.54), respectively. NTCP_ex_ was a median of 1.77 (range 1.60–1.99) times as high as the NTXP_ref_. In a logarithmic comparison, the risk of RN is enhanced by a factor of median 2.00 (range 1.66–2.35). The defined constraints for the organs at risk were feasible.

**Conclusion:**

When considering the potential protective effect of BEV, which we hypothesized might reduce the risk of RN by approximately two-fold, achieving isotoxicity with the proposed dose-escalated experimental plan for the PRIDE trial seems feasible.

## Highlights

• The presented dose escalation strategy doubles the risk of radiation necrosis.
• While Literature suggests that Bevacizumab can halve its risk.
• Dose constraints of organs at risk were adhered to.
• Therefore, isotoxicity might be achieved in the upcoming PRIDE trial.

## 1 Introduction

The current standard of care for glioblastoma involves performing a maximum safe resection followed by radiochemotherapy delivered at a dose of 60 Gy in 30 fractions or 40 Gy in 15 fractions depending on the patient́s age and clinical condition [1], [2], [3], [4]. Concurrent and sequential chemotherapy is administered following either the EORTC 26981/22981 or the NOA-09 regimen, depending on the methylguanine methyltransferase (MGMT) promoter methylation status [1], [5]. Despite the aggressive nature of the current treatment regimens, the overall survival (OS) of patients with glioblastoma remains poor, emphasizing the need for novel and improved treatment strategies.

A major challenge in the treatment of glioblastoma is the insufficiency of the standard radiation dose to effectively control the tumor. Supporting this notion is the observation of many publications that 75–93 % of recurrences/progressions manifest within the primary tumor area [6], [7], [8], [9], [10]. To address this concern, several trials have explored dose escalation concepts, with some studies reporting favorable outcomes in terms of OS and progression-free survival (PFS) [11], [12], [13], [14], [15], [16], [17], [18], [19]. However, these benefits are mostly accompanied by an elevated risk of treatment-related toxicity such as radiation necrosis (RN).

**Fig. 1:**
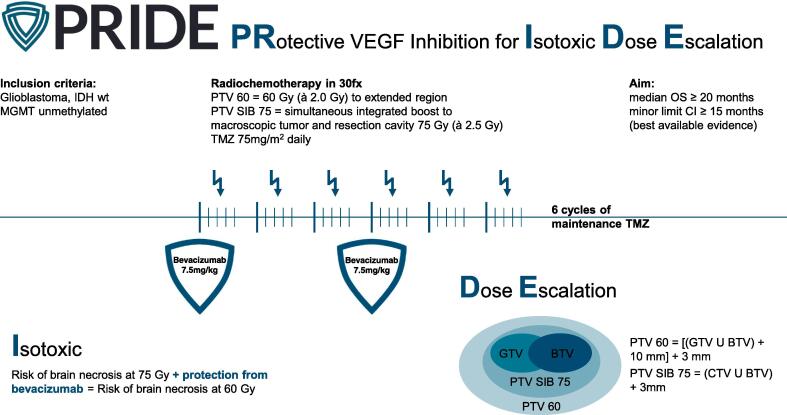
Graphical overview of the concept of the upcoming PRIDE trial *(BTV = biological target volume; CI = confidence interval; fx = fractions; GTV = gross tumor volume; IDH wt = isocitrate dehydrogenase wild type; MGMT = methylguanine methyltransferase; OS = overall survival; PTV = planning target volume; SIB = simultaneous integrated boost; TMZ = temozolomide)*.

To address this issue of increased RN risk, the PRIDE trial (“Protective VEGF Inhibition for Isotoxic Dose Escalation in Glioblastoma”; NCT05871021; NOA-28; ARO-2022-12) aims to incorporate the vascular endothelial growth factor (VEGF) inhibitor bevacizumab (BEV) into dose-escalated radiation therapy (RT), with doses up to 75 Gy (Fig. 1). BEV is known to be effective in treating steroid-refractory edema and RN [20], [21]. Its protective potential during re-irradiation treatment was shown in a study by Fleischmann et al, reducing the one-year risk of RN and symptomatic edema from 54.1 % to 23.9 %, p = 0.013; the percentage of patients with RN was substantially reduced in patients receiving BEV compared to patients not receiving BEV (4.8 % vs. 13.5 %; p = 0.078) [22]. Assuming that the risk of RN doubles with the proposed dose escalation schedule and that the protective effects of BEV are consistent in primary RT, dose-escalated RT with concurrent BEV could potentially achieve isotoxicity compared to standard RT, while potentially improving PFS and possibly extending OS.

**Fig. 2:**
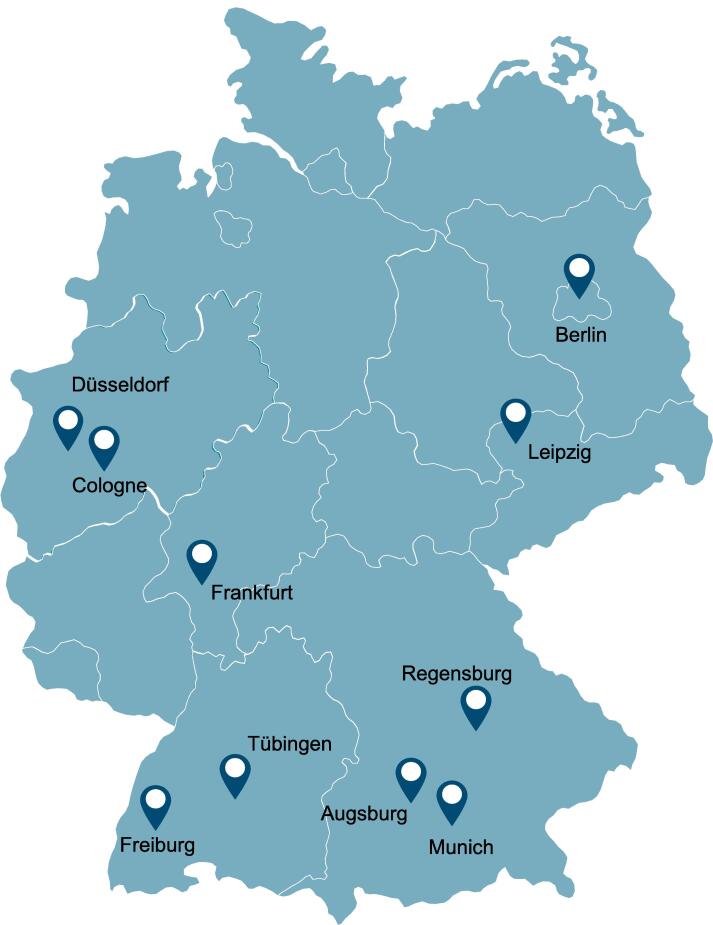
A map of Germany depicting the ten centers, who will be participating in the PRIDE trial.

**Fig. 3:**
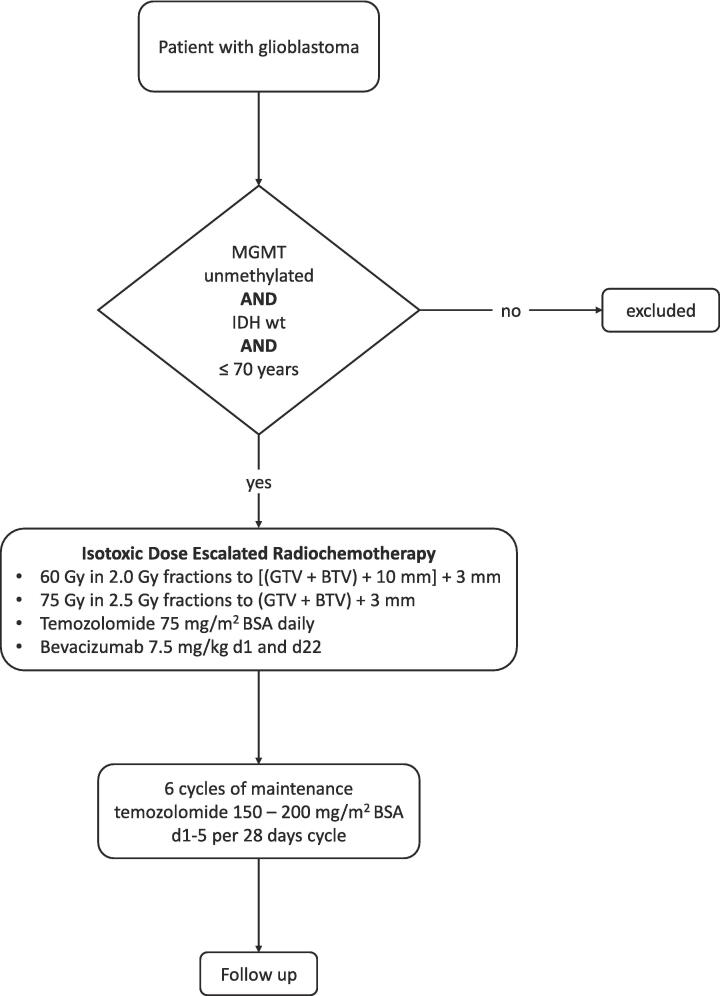
A Flow-Chart showing the inclusion and treatment plan for patients in the upcoming PRIDE trial *(BTV = biological target volume; GTV = gross tumor volume; IDH wt = Isocitrate dehydrogenase wild type; MGMT = methylguanine methyltransferase)*.

The PRIDE trial is designed as a, single-arm, open-label, non-randomized, multicenter phase IIa study. It is intended to include a total of 146 patients from ten different centers as depicted in Fig. 2. The cohort for the PRIDE trial will consist of patients with glioblastoma who meet the following criteria: isocitrate dehydrogenase wild-type (IDH wt), MGMT non-methylated, aged between 18 and 70 years, and clinically eligible to receive temozolomide (TMZ) and BEV. The complete inclusion and exclusion criteria are shown in Table 1, while a visual representation of the patient selection process is provided in Fig. 3. The primary endpoint of the PRIDE trial is OS, which will be the main measure of treatment effectiveness. The trial also includes several secondary endpoints, including treatment safety and tolerability of the treatment, PFS, quality of life assessment, and evaluation of cognitive function. In addition to these endpoints, there is a translational exploratory objective in the study to validate a 4-micro-ribunucleoid-acid (4-miRNA) signature-based risk subgroup classification [23], [24]. This objective aims to investigate the potential of a specific 4-miRNA signature in predicting treatment response or prognosis. Recruitment for the study is planned to start on October 1st, 2023, and continue until October 1st, 2024. The end of the study is scheduled for October 1st, 2026 ensuring a minimum follow-up period of 24 months for the enrolled patients.

**Table 1:** The Inclusion and exclusion criteria of the PRIDE-trial (*AST = aspartate aminotransferase; ALT = alanine aminotransferase; IDH = Isocitrate dehydrogenase; MGMT = methylguanine methyltransferase; ECOG = Eastern Cooperative Oncology Group; ULN = upper limit of normal; NYHA = New York Heart Association; NCI-CTC = National cancer institute common terminology criteria)*.

**Main Inclusion Criteria:** - **IDH wild-type, MGMT unmethylated glioblastoma patients** - Informed consent - **Age ≥18 and ≤70 years,** smoking or non-smoking, of any ethnic origin - **ECOG score of 0–2** - Neutrophil counts >1500/μl; Platelet counts >100.000/μl; Hemoglobin >8 g/dl; Serum creatinine <1.5-fold upper limit of normal (ULN); Bilirubin, AST or ALT <2.5-fold ULN unless attributed to anticonvulsants; Alkaline phosphatase <2.5-fold ULN - **Adequate contraception** - Serum creatinine ≤1.5 x ULN AND patients with urine dipstick for proteinuria <2+. Patients with ≥2 + proteinuria on dipstick urinalysis at baseline should show urine protein to creatinine ratio ≤1 or should undergo a 24-hour urine collection and must demonstrate ≤1 g of protein in 24 h **Main Exclusion Criteria:** - **Evidence of recent hemorrhage** on postoperative MRI of the brain. However, patients with clinically asymptomatic presence of hemosiderin, resolving hemorrhagic changes related to surgery, and presence of punctate hemorrhage in the tumor are permitted entry into the study - Subjects on **any drug suspected to interfere with bevacizumab** at the time of study inclusion - **Immuno-compromised patients**, including known seropositivity for human immunodeficiency virus (HIV) - Known **hypersensitivity** to any component of the investigational drugs or excipients (allergy to or other intolerability of bevacizumab or excipients) - Any **other significant medical illness** or medically significant laboratory finding that would, in the investigator’s judgement, make the patient inappropriate for this study, or would increase the risk associated with the patients’ participation in the study - **Incapability to undergo MRI** - - **Prior treatment with bevacizumab for** any indication	**Bevacizumab related Exclusion Criteria** - **Contraindication and/or hypersensitivity to bevacizumab** or its excipients. For details check the Summary of Product Characteristics - **Significant cardiovascular disease** defined as congestive heart failure (NYHA Class II, III, IV), unstable angina pectoris, or myocardial infarction within 6 months prior to enrolment - **Inadequately controlled hypertension** (de-fined as a blood pressure of >150 mmHg systolic and/or >100 mmHg diastolic on medication), or any prior history of hypertensive crisis or hypertensive encephalopathy - **History of stroke or transient ischemic attack** within 6 months prior to enrolment - **Significant vascular disease** (e.g. aortic aneurysm, aortic dissection or recent peripheral arterial thrombosis) within 6 months prior to enrolment - **Evidence or history of recurrent thromboembolism** (>1 episode of deep venous thrombosis/peripheral embolism) during the past 2 years - **Evidence of bleeding diathesis or coagulopathy** (in the absence of therapeutic anticoagulation) - **Chronic daily intake of aspirin** >325 mg/day or clopidogrel >75 mg /day - **History of intracranial abscess** within 6 months prior to inclusion - **History of abdominal or tracheo-oesophageal fistula, gastrointestinal perforation, or intra-abdominal abscess** within 6 months prior to study enrolment - **History of ≥grade 2 hemoptysis** according to NCI-CTC criteria within 1 month prior to inclusion - - **Serious non-healing wound, ulcer or bone fracture**

The primary objective of this dosimetric feasibility study is to conduct a comparative analysis between the standard treatment approach and the experimental treatment protocol which will be utilized in the PRIDE trial. The results obtained from this study will contribute to determining the feasibility of the proposed treatment concept for the upcoming phase II trial.

## 2 Material and methods

For this dosimetric feasibility study, a total of ten patients, who had previously undergone treatment for glioblastoma, were selected from the internal clinical database MOSAIQ® (Elekta, Stockholm, Sweden). The contrast-enhanced T1 and T2 FLAIR weighted magnet resonance imaging (MRI) sequences, the planning computed tomography (CT) scans, and the *O*-(2-18F-fluoroethyl)-L-tyrosine (FET) positron emission tomography (PET) scans from the primary treatment were utilized to generate two distinct RT treatment plans. The first plan, serving as the reference, was generated following the ESTRO-EANO contouring guidelines of 2023 [25]. The second plan was a modified plan generated in accordance with the specific study guidelines outlined for the PRIDE trial.

### 2.1 Target definition

The gross tumor volume (GTV) was created by delineating the contrast-enhanced tumor and the resection cavity in the T1 MRI sequence. Highly suspicious T2 FLAIR enhancements were also included in the GTV. In addition to the GTV, a biological tumor volume (BTV) was generated using the target-to-background ratio (TBR) threshold of 1.8, based on the FET PET scan with the assistance of nuclear medicine experts (further details provided in the subsequent section) [26], [27]. The union of the GTV and the BTV was defined as GTVu. In the reference plan, the clinical target volume (CTV) receiving 60 Gy (CTV60) was generated by expanding the GTVu with a 15 mm margin, following the 2023 ESTRO-EANO contouring guidelines, and anatomical adjustements [25]: The contour was cropped at the skull, the falx cerebri, the tentorium cerebelli, the optic nerves, the optic chiasm and the brainstem allowing no overlap (except in the case of direct/midbrain involvement); at the ventricles an overlap of 5 mm was allowed. In the experimental plan the CTV receiving 60 Gy (CTV60ex), was created by expanding the GTVu with a 10 mm margin (5 mm less than in the reference plan); the anatomical adjustments were equivalent to the CTV60. To generate the corresponding planning target volumes (PTV) the CTV60 and the CTV60ex were expanded with a 3 mm margin resulting in the PTV60 and the PTV60ex, respectively, without further modifications. In the experimental treatment plan, an additional PTV was created specifically for the dose escalation to 75 Gy (PTV75); it was generated by expanding the GTVu with a 3 mm margin, with no further adjustments. If the PTV75 was adjacent to or infiltrating critical structures, such as the brainstem, the chiasm or the optic nerves, a PTV75opt was generated by subtracting the mentioned organs at risk (OAR) with a safety margin of 5 mm from the PTV75. All target volumes were delineated by three radiation oncology consultants, who reached a consensus on the contours of the structures.

### 2.2 Definition of BTV on FET PET

BTV on FET PET was defined as recommended in the joint nuclear medicine/neuro-oncology procedural guidelines on amino acid PET. Summation PET images acquired 20–40 min post injection were used [26]. The mean background activity was assessed using crescent-shaped regions-of-interest in the brain hemisphere contralateral to the target lesion, as previously established [27]. BTV was semi-automatically delineated using a cut-off threshold of 1.8× background activity (target-to-background ratio, TBR); all voxels above a TBR of 1.8 were included into the BTV. In case of multifocal disease, all BTVs were summed up. Each BTV was individually controlled for potential spill-in uptake of adjacent structures, such as vessels and the skull, and manually adjusted if necessary.

### 2.3 Dose planning

All plans were generated by a medical physicist using the inverse planning software MONACO® by Elekta (Stockholm, Sweden), which utilizes a Monte Carlo dose calculating algorithm. The prescription details for the experimental and reference plan are presented in Table 2. OAR constraints were used as given per ESTRO-EANO guideline 2023 by Niyazi et al with slight adaptations and summarized in Table 3 [25]. To optimize the dose distribution and achieve a balance between adequate target coverage and minimizing radiation exposure to the OAR, the medical physicist was encouraged to employ non-coplanar arcs.

**Table 2:** Prescription Details for Experimental Plan and Reference Plan *(PD = prescription dose; GTVu = the union of the gross tumor volume and the biological tumor volume; PTV60, PTV60ex = the planning target volume of the reference plan and the experimental plan prescribed with 60 Gy; PTV75 = the planning target volume of the experimental plan prescribed with 75 Gy; PTV75opt = the optimized PTV75 for volumes adjacent to the brainstem or the optic tract; D98, D50, D2 = the dose covering 98 %, 50 % and 2 % of the volume, standing for the near minimal, mean and near maximal dose, respectively)*.

Structure	Parameter	Per Protocol	Acceptable Variation
***Experimental Plan***			
GTVu	D98	71.25 Gy	60 Gy (when GTVu directlyadjacent to Brainstem)
PTV60ex	D98	57 Gy (95 % of PD)	54 Gy (90 % of PD)
PTV75/PTV75opt	D98	71.25 Gy (95 % of PD)	67.5 Gy (90 % of PD)
	D2	≤80.25 Gy (≤107 % of PD)	≤82.5 Gy (≤110 % of PD)
	D50	100 % of PD	±2%

***Reference Plan***			
	D98	57 Gy (95 % of PD)	54 Gy (90 % of PD)
PTV60	D2	≤64.2 Gy (≤107 % of PD)	≤66 Gy (≤110 % of PD)
	D50	100 % of PD	±2%

**Table 3:** Organs at risks (OAR) and their dose constraints *(PTV = planning target volume; V40, V45 = the percentage of brain volume covered by 40 Gy and 45 Gy; D0.03 cc = the dose covering 0.03 cc; Dmean = the mean dose received by the volume; EUD = equivalent uniform dose; PRV = planning organ at risk volume)*.

Organ at risk	Parameter(s)	Per Protocol	Acceptable Variation	May compromise PTV coverage?	Comment
Skin	D0.03 cc	As low as reasonably achievable		No	Structure between the external contour and the external contour reduced by 3 mm
Lacrimal Glands	D0.03 cc	25 Gy	40 Gy	No	
	Dmean	≤25 Gy	all	No	
Eye Lenses	D0.03 cc	6 Gy	10 Gy	No	
Optic Nerves	D0.03 cc	54 Gy	55 Gy	Yes	
Optic Nerves PRV	D0.03 cc	56 Gy	60 Gy	Yes	Optic nerves expanded by 3 mm
Optic Chiasm	D0.03 cc	54 Gy	55 Gy	Yes	
Optic Chiasm PRV	D0.03 cc	56 Gy	60 Gy	Yes	Optic chiasm expanded by 3 mm
Pituitary Gland	D0.03 cc	40 Gy	54 Gy	No	
Brainstem	D0.03 cc	54 Gy (preferred, if PTV coverage is not compromised)	60 Gy	Yes	
		56 Gy(in other cases)			
Brainstem Centre	D0.03 cc	54 Gy	56 Gy	Yes	Brainstem reduced by 3 mm
Cochleae	D0.03 cc	40 Gy	60 Gy	No	
	Dmean	≤45 Gy	all	No	
Eyes	D0.03 cc	54 Gy	all	No	
Brain	EUD, Dmean, V45, V40	As low as reasonably achievable		No	Brain tissue excluding cavernous sinuses, brainstem, optic chiasm, optic nerves, pituitary, mammillary bodies, meckel’s caves and GTVu

### 2.4 Equivalent uniform dose (EUD)

The EUD concept introduced by Niemierko et al. was employed to evaluate and compare the quality of the reference and experimental treatment plans [28]. The EUD represents the radiation dose that would result in the same clinical effect as achieved by the actual heterogeneous dose distribution, assuming an idealized homogeneous dose distribution [29], [30], [31], [32].

The EUD is calculated by using the formula depicted below, which takes into account the irradiated partial volumes (*V_i_*) and doses (*D_i_*) in each bin, and the volume-effect parameter *a* (a ∊ [1… ∞[). For *a*->∞ and *a* = 1 the EUD would be equivalent to the maximum dose (Dmax) and the mean dose (Dmean), respectively.

$$\mathrm{EUD}={\left(\sum_{i=0}^{n}{V_{i}D_{i}}^{a}\right)}^{\frac{1}{a}}$$

### 2.5 Normal tissue complication probability (NTCP) model

The NTCP model developed by Niyazi et al. was applied for comparison of the RN risk assuming no differences between photon and proton dose distributions, since the effects appear to be independent of the linear energy transfer [33], [34]. The NTCP model used in this study is based on the EUD of the brain (pure brain tissue excluding cavernous sinuses, brainstem, optic chiasm, optic nerves, pituitary, mammillary bodies, Meckel’s caves and GTVu) with a = 9 and converted to 30 fractions:

$$\mathrm{NTCP}=\frac{1}{1+{\left(\frac{55.5Gy}{{\mathrm{EUD}}_{9}}\right)}^{10}}\;\;\;$$

As we hypothesize that BEV can reduce the risk of RN by a factor of 2–3, similar as what has been shown for patients receiving reirradiation [22], we aim to achieve a similar factor for the ratio of the two NTCP values (NTCP_ref_, NTCP_ex_: the NTCP of the reference and the experimental treatment plan, respectively):

$${\mathrm{NTCP}}_{\mathrm{Ratio}}=\frac{{\mathrm{NTCP}}_{\mathrm{ex}}}{{\mathrm{NTCP}}_{\mathrm{ref}}}$$

A more precise estimation to assess the increased risk (IR) of the experimental plan was performed with the following logarithmical formula:

$$\mathrm{IR}=\frac{\ln(1-{\mathrm{NTCP}}_{\mathrm{ex}})}{\ln(1-{\mathrm{NTCP}}_{\mathrm{ref}})}$$

A value of “1”, “2” or “3” would mean that the risk of RN is identical, increased by a factor two, or by a factor three, respectively, for the experimental plan compared to the reference plan.

### 2.6 Plan comparison

The ProKnow® cloud-based platform (Elekta, Stockholm, Sweden), was employed for the comparison and analysis of both treatment plans. The anonymized images, registrations, structure sets, and dose distributions of the reference and experimental plan were uploaded to the platform for further analysis. Using the prescription and constraints provided in Table 2, Table 3, scorecards were created enabling the assessment and comparison of plan quality.

## 3 Results

**Fig. 4:**
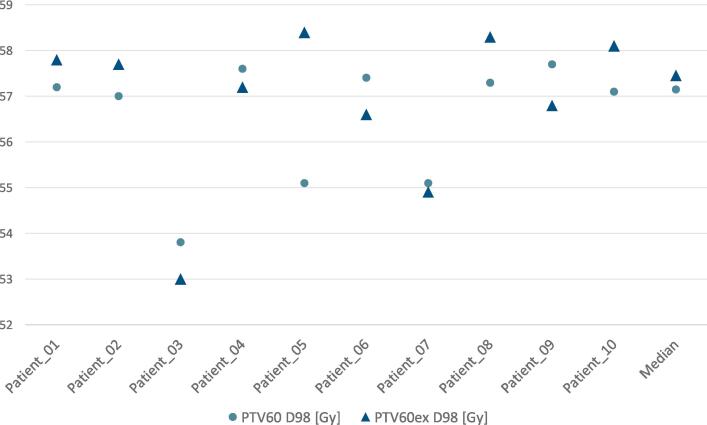
A diagram comparing the D98 (vertical axis in Gy) between the standard plan (PTV60) and the experimental plan (PTV60ex) of each patient.

**Fig. 5:**
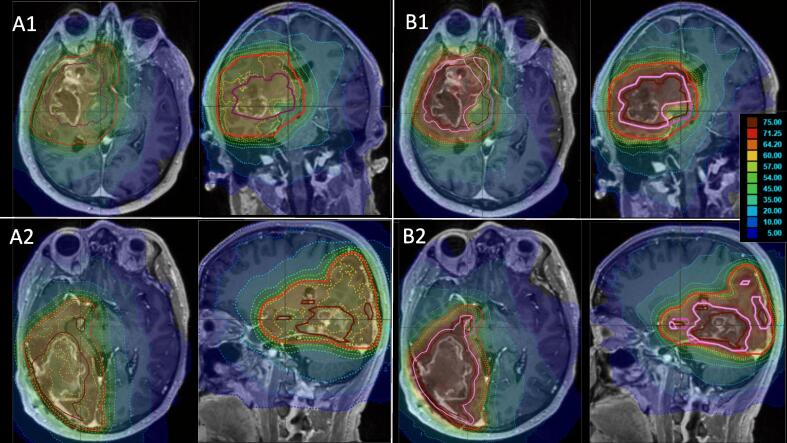
MRI images including dose distribution of two patients, which required a PTVopt due to adjacency to the brainstem and/or optical tract; A1 + 2 are reference plans, B1 + 2 are the experimental plans; A1/B1 is Patient_3; A2/B2 is Patient_7; the red lines is are PTV60 or PTV60ex, dark violet the GTVu, magenta the PTV75, and pink the PTV75opt *(MRI = magnet resonance imaging; GTVu = the union of the gross tumor volume and the biological tumor volume; PTV60, PTV60ex = the planning target volume prescribed with 60 Gy of the reference plan and of the experimental plan; PTV75 = the planning target volume prescribed with 75 Gy of the experimental plan; PTV75opt = the optimized PTV75 for patients adjacent to the brainstem or the optic tract)*.

In the reference plan, median (range) D98, D2 and D50 (dose which is covered by 98 %, 2 % or 50 %, representing the near-minimal, near-maximal and mean dose, respectively) of PTV60 was 57.1 Gy (53.8–57.7 Gy), 62.8 Gy (62.2–66.6 Gy) and 60.4 Gy (59.9–62.8 Gy), respectively. In the experimental plan median (range) D98 of GTVu was 73.9 Gy (58.5–74.6 Gy), D98 of PTV60ex was 57.4 Gy (53.0–58.4 Gy), and D98, D2 and D50 of PTV75 was 71.5 Gy (55.6–72.5 Gy), 78.1 Gy (77.5–78.7 Gy) and 74.8 Gy (74.4–75.9 Gy). D98 of PTV60ex was 57.4 Gy (range 53.0–58.4 Gy), and D98, D2 and D50 of PTV75 was 71.5 Gy (range 55.6–72.5 Gy), 78.1 Gy (range 77.5–78.7 Gy) and 74.8 Gy (range 74.4–75.9 Gy). The comparison of D98 between both plans is highlighted in Fig. 4. In Patient_03 and Patient_07 a PTV75opt (marked in Table 3 with an asterisk) was used due to the PTV75 being adjacent to the brainstem and/or the optic tract. The plans of Patient_03 and Patient_07 are depicted in Fig. 5. Table 4 lists the mentioned values for each patient.

**Table 4:** The dose values of the target volumes for both plans of every patient: the green values are completely within protocol, the yellow values are acceptable variations, the orange values are above or below the margin of acceptance; the ranges are shown in Table 2. The numbers in the brackets with the asterisk (*) show the corresponding numbers of the PTV75opt, which was generated in this cases due to adjacency to critical organs at risk *(D98, D50, D2 = the dose covering 98 %, 50 % and 2 % of the volume standing for the near minimal, mean and near maximal dose, respectively; GTVu = the union of the gross tumor volume and the biological tumor volume; PTV60, PTV60ex = the planning target volume prescribed with 60 Gy of the reference plan and of the experimental plan; PTV75 = the planning target volume prescribed with 75 Gy of the experimental plan)*.

**Fig. 6:**
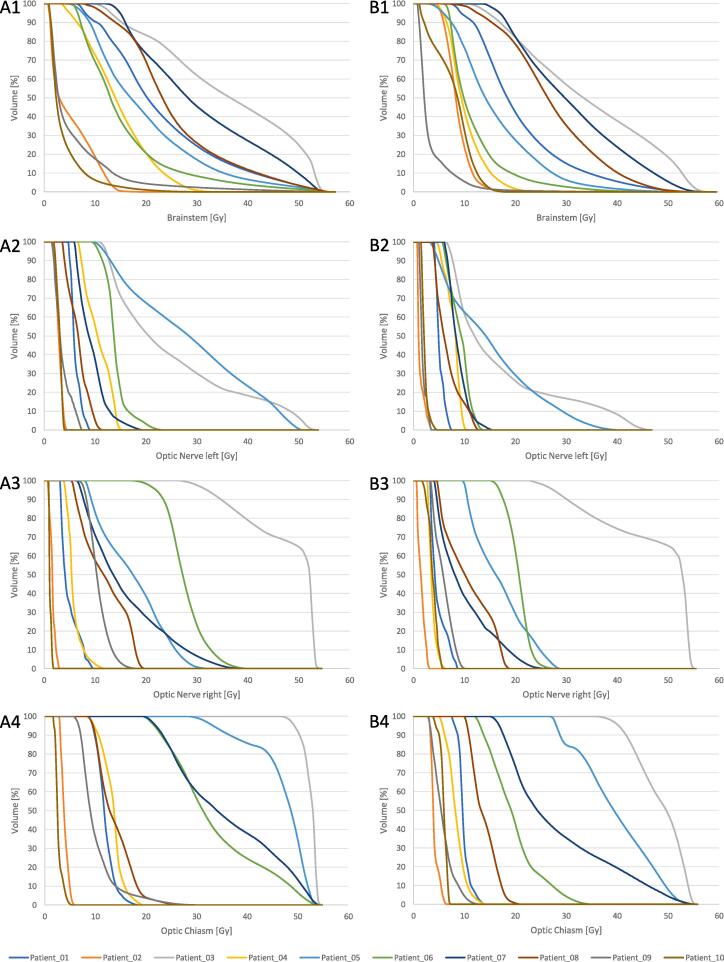
The dose-volume histograms (DVH) of the brainstem (A1, B1), the optic nerves (A2-3, B2-3) and the optic chiasm (A4, B4); A1-4 are from the reference plan, B1-4 from the experimental plan.

Median (range) D0.03 cc (dose covering 0.03 cc, representing the maximum dose in a measurable volume) was 55.1 Gy (17.8–56.0 Gy) and 48.5 Gy (17.3–57.5 Gy) for the brainstem, and 45.1 Gy (13.8–53.7 Gy) and 32.2 Gy (10.2–53.8 Gy) for the brainstem center (brainstem minus 3 mm inner margin) in the reference and the experimental plan, respectively. The exact values of each patient and plan of the critical OARs are listed in Table 5. The corresponding dose-volume histograms are shown in Fig. 6. The dose values for all OARs are listed in the Supplement Table.

**Table 5:** The dose exposure of the organs at risk (OAR): the green values are completely within protocol, the yellow values are acceptable variation, the orange values are above or below the margin of acceptance, the non-colored values do not have any specific constraints; the accepted ranges are shown in Table 3*(D0.03 cc = the dose covering 0.03 cc; Dmean = the mean dose received by the volume; Ref = the reference plan; Ex = the experimental plan)*.

**Fig. 7:**
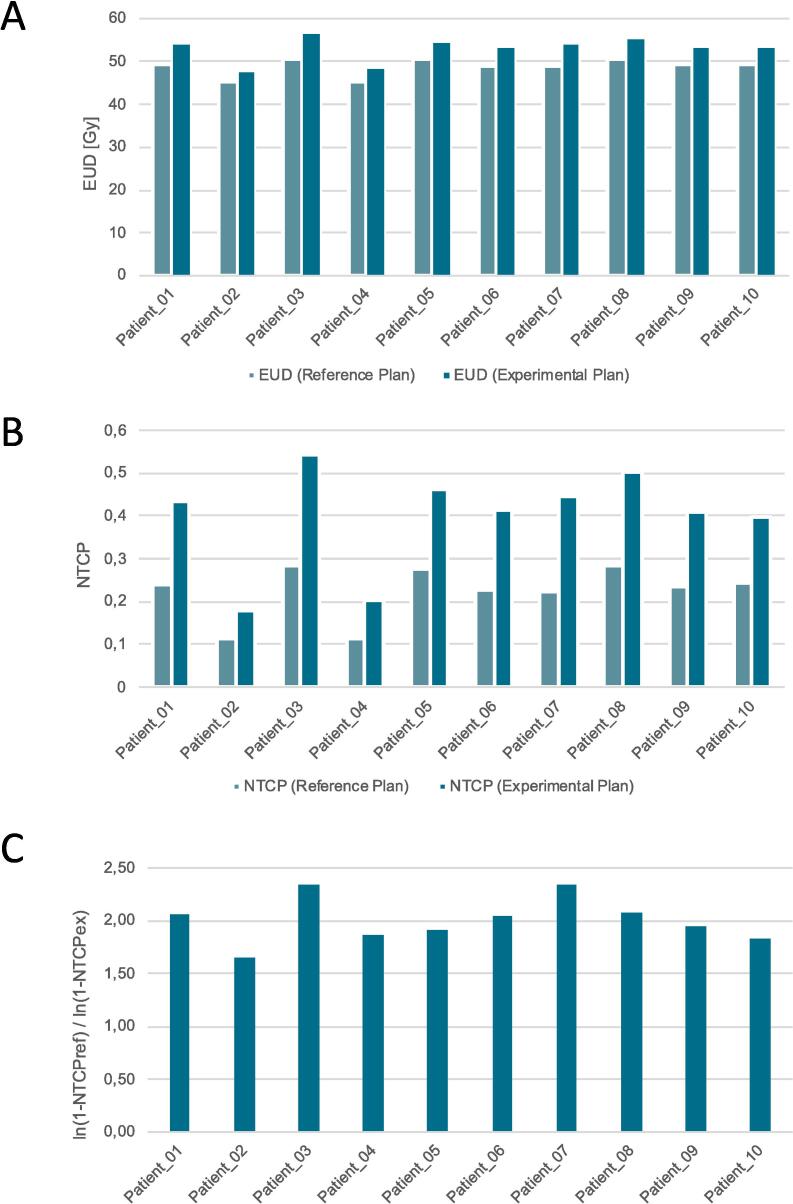
Diagrams depicting the EUD (A) and NTCP (B) values of each patient in the reference and experimental plan. The third diagram (C) shows the results of the logarithmical comparison mentioned in the methods section (*EUD = equivalent uniform dose; NTCP = normal tissue complication probability; ref = the reference plan; ex = the experimental plan)*.

Median (range) volume of GTVu, PTV60, PTV60ex and PTV75 were 54.4 cc (6.6–97.8 cc), 275.9 cc (97.9–370.9 cc), 204.7 cc (62.1–297.2 cc) and 88.4 cc (14.7–149.8 cc). Median (range) V40 and V45 of the brain were 21.7 % (10.0–29.4 %) and 19.5 % (9.0–26.6 %) for the reference plan and 15.1 % (5.8–25.8 %) and 13.5 % (5.2–22.7 %) for the experimental plan, respectively. Median (range) EUD of the brain was 49.5 Gy (45.1–50.6 Gy) and 54.0 Gy (47.6–56.4 Gy) for the reference and the experimental plan, respectively. The NTCP values were calculated as described above: Median (range) NTCP_ref_ and NTCP_ex_ were 0.24 (0.11–0.29) and 0.42 (0.18–0.54), respectively. NTCP_ex_ was a median of 1.77 (range 1.60–1.99) times as high as the NTCP_ref_. The logarithmical comparison resulted in a median value of 2.00 (range 1.66–2.35). For the exact values of each patient for each plan including the comparisons see Table 6, and for a graphical visualization of the EUD and NTCP for both plans see Fig. 7.

**Table 6:** The parameters related with the risk of radiation necrosis; the numbers in the brackets with the asterisk (*) show the corresponding volumes of the PTV75opt, which was generated in this cases due to adjacency to critical organs at risk *(EUD = equivalent uniform dose; NTCP = normal tissue complication probability; Ref = the reference plan; Ex = the experimental plan; GTVu = the union of the gross tumor volume and the biological tumor volume; PTV60, PTV60ex = the planning target volume prescribed with 60 Gy of the reference plan and of the experimental plan; PTV75 = the planning target volume prescribed with 75 Gy of the experimental plan; V40, V45 = the percentage of brain volume covered by 40 Gy and 45 Gy).*

Patient ID	GTVu [cc]	PTV60 [cc]	PTV60ex [cc]	PTV75 [cc]	Brain V40 [%]		Brain V45 [%]		EUD [Gy]		NTCP		$\frac{{\mathrm{NTCP}}_{\mathrm{ex}}}{{\mathrm{NTCP}}_{\mathrm{ref}}}$	$\frac{\ln(1-{\mathrm{NTCP}}_{\mathrm{ex}})}{\ln(1-{\mathrm{NTCP}}_{\mathrm{ref}})}$
					Ref	Ex	Ref	Ex	Ref	Ex	Ref	Ex		
Patient_01	66.3	256.1	208.6	102.8	16.7	15.5	15.1	13.8	49.5	54.0	0.24	0.43	1.80	2.06
Patient_02	6.6	97.9	62.1	14.7	10.6	6.0	9.4	5.3	45.1	47.6	0.11	0.18	1.60	1.66
Patient_03	90.6	370.8	300.1	139.3 (128.3)*	29.4	25.8	26.6	22.7	50.6	56.4	0.28	0.54	1.91	2.35
Patient_04	11.4	120.0	78.3	22.9	10.0	5.8	9.0	5.2	45.2	48.4	0.11	0.20	1.78	1.87
Patient_05	38.4	281.2	200.8	71.1	27.2	19.8	24.5	17.7	50.4	54.7	0.28	0.46	1.67	1.92
Patient_06	56.7	291.7	216.6	92.5	20.3	14.1	18.5	12.6	49.1	53.6	0.23	0.41	1.81	2.05
Patient_07	97.8	370.9	297.8	149.8 (148.2)*	22.4	18.2	20.1	15.8	49.0	54.3	0.22	0.45	1.99	2.33
Patient_08	88.9	359.6	279.2	139.7	29.3	24.2	26.0	20.9	50.6	55.6	0.29	0.50	1.76	2.07
Patient_09	52.2	270.6	200.4	84.4	21.0	13.9	19.0	12.6	49.4	53.5	0.24	0.41	1.72	1.94
Patient_10	13.5	203.1	136.9	32.9	22.7	14.8	20.3	13.2	49.5	53.3	0.24	0.40	1.65	1.83
**Median**	**54.4**	**275.9**	**204.7**	**88.4**	**21.7**	**15.1**	**19.5**	**13.5**	**49.5**	**54.0**	**0.24**	**0.42**	**1.77**	**2.00**

## 4 Discussion

Historically, the primary goal of RT for glioblastoma has been to effectively treat the extensive non-visible tumor cell population, given the infiltrative nature of glioblastoma. In fact, up to the 1990 s glioblastoma was treated with whole-brain RT in addition to RT to the tumor volume or resection cavity [35]. With the advancement of imaging techniques, the size of the treatment volumes gradually decreased. The 2016 ESTRO-ACROP guideline recommended expanding the GTV with 2.0 cm and optionally including the entire visible edema in the T2 sequence of the MRI [36]. Despite the considerable reduction of the treatment volume compared to the historical whole-brain RT, volumes were still quite large. The introduction of advanced imaging techniques, such as amino acid PET (e.g. FET PET), which enables the delineation of a Biological Tumor Volume (BTV), offered the potential for further refinement of treatment volumes [37]. Several studies investigating FET-based planning and recurrence analyses have reported on the reduction of treatment margins. For instance, a recurrence pattern analysis by Fleischmann et al. described a feasible reduction in the margin to 1.5 cm, while Laack et al. went even further by reducing the margin to 1.0 cm [38], [39]. Following these observations, the recent ESTRO-EANO guideline from 2023 recommends a GTV-to-CTV-margin of 1.5 cm (1.0–1.5 cm for “molecularly defined” tumors); additionally, the edema is no longer included in the CTV [25]. In addition to margin reduction, PET-based planning has been explored as a strategy for dose escalation in glioblastoma treatment. Notably, Piroth et al and Kim et al have conducted studies exploring the concept of escalating the radiation dose to 72 Gy and 75 Gy, respectively [16], [19].

Piroth et al. implemented dose escalation guided by PET imaging in their study. Their hypothesis was that utilizing FET PET could optimize coverage and allow regional dose escalation in the areas containing viable tumor tissue. They conducted a phase II trial involving 22 patients, administering radiotherapy through MRI- and FET PET-guided integrated-boost intensity-modulated radiotherapy. Interestingly, the authors concluded that their concept did not yield an OS benefit. However, they noted the significant presence of MGMT unmethylated patients who underwent subtotal tumor resection, a factor the authors acknowledged but did not take into account in their analysis. In contrast to the planned PRIDE study dose-escalation was done up to 72 Gy and the resection cavity was excluded from the volume which received dose-escalation [16]. Kim et al. conducted a study investigating dose-escalated radiotherapy in glioblastoma patients recruited between 2016 and 2018 [19]. In this single-arm phase II study, dose escalation to hypercellular/hyperperfused tumor regions showed promising improvements in OS, along with positive impacts on short-term neurocognitive function, symptomatic burden, and quality of life. This study enrolled 26 patients and employed MRI along with 11C-Methionine (MET) PET imaging to delineate at-risk brain areas. Two patients exhibited late grade 3 neurologic toxicity. Among the cohort, only five out of 22 patients experienced central-to-in-field recurrence. Notably, the 1-year survival rate stood at 92 %, with a median OS of 20 months observed among the 13 patients who had received a radiation boost targeting both hypercellular and hyperperfused tumor regions [19]. The most recently published trial exploring dose escalation is the SPECTRO GLIO trial using a slightly different approach by utilizing MRI spectroscopy to define the region which should receive dose escalation to 72 Gy [40]. Median OS and median PFS was 22.6 months and 22.2 months, and 8.6 and 7.8 months, in the cohort with and without dose escalation, respectively. Despite the lack of a clear benefit for the dose-escalated cohort, there are still several reasons why PRIDE could potentially be successful. Firstly, the cohort, viewed through the lens of the WHO 2021 criteria, exhibits heterogeneity with partially unknown IDH status and a mixed MGMT methylation status [41]. Additionally, MR spectroscopy might be somewhat less suitable for boost delineation compared to FET PET. An intriguing aspect of the study's results is the high OS outcome observed in the control cohort, as noted by Shu et al. in a letter [42], and the absence of a difference in the rate of radiation necrosis [40]. Compared to these results, Tsien et al. (2019) presented encouraging outcomes regarding dose-escalation in their study [11]. The primary aim of this phase I trial was to demonstrate the feasibility and assess the toxicity of dose-escalated radiotherapy alongside chemotherapy among patients diagnosed with primary supratentorial glioblastoma. A cohort of 209 patients were included in this study, showcasing the viability of administering radiotherapy doses exceeding the standard 60 Gy, concurrently with chemotherapy for primary glioblastoma. Importantly, the study indicated an acceptable risk profile for late central nervous system toxicity. Despite not being explicitly designed for this outcome, the higher dose arms exhibited a noteworthy extension in median overall survival (OS) [11]. In a study by Laack et al. 75 patients with a FET-PET based dose-escalated RT were compared to 139 patients treated with standard RT at the same institution [38]. Median PFS was significantly improved by dose-escalation (8.7 vs. 6.6 months; p = 0.017), OS was non-significantly improved in MGMT unmethylated patients (16.0 vs. 13.5 months; p = 0.13) and significantly improved in MGMT methylated patients (35.5 vs 23.3 months; p = 0.049) [38].

The PRIDE trial takes advantage of PET-based planning techniques and implements a reduction in the GTV-to-CTV margins to 1.0 cm. Simultaneously, the dose administered to the morphologically and biologically defined tumor volume is escalated to 75 Gy delivered in 30 fractions. The idea of this concept is to minimize radiation exposure to potentially unaffected brain tissue while intensifying treatment to the true tumor volume, aiming for a more aggressive approach. The addition of BEV is intended to mitigate the potentially increased RN risk from dose escalation.

The feasibility of the PRIDE concept relies on ensuring that the increased risk of RN from dose-escalated RT does not exceed the protective potential of BEV. The logarithmical NTCP-comparison of the experimental and reference plan led to a median value of 2.00 (range 1.66–2.35), meaning the risk of RN is increased by a median factor of 2 in the dose-escalated RT compared to the reference plan. Having the described protective effect of BEV with a factor of 2–3 in mind, the concept appears to be feasible. Furthermore, the concept of margin reduction may provide additional benefits to OARs despite of the dose escalation: Upon reviewing the dose values in Table 5 and examining the dose-volume histograms (DVH) graphs in Fig. 6, it becomes evident that the critical OARs are exposed to lower levels of radiation in the majority of experimental plans compared to their corresponding reference plans. There are, however, two exceptions to this trend, specifically in the cases of Patient_03 and Patient_07. In both cases, the GTVu is either infiltrating (Patient_03) or adjacent (Patient_07) to critical structures such as the brainstem and/or the optic tract (see Fig. 5). Although both patients (Patient_03 and Patient_07) exceeded the constraints given by the protocol for the brainstem and partly the optic system, it is important to note that they did neither exceed their acceptance range nor the constraints for the brainstem center (Table 5). The dose prescription requirements (Table 2) were found to be outside of the acceptance range in two cases (Table 4): in Patient_03 due to the aforementioned circumstances in the experimental plan, and Patient_01 only in the reference plan. In the reference plan for Patient_01, the elevated dose peaks were observed due to attempts to achieve a higher dose gradient. However, it is worth noting that in the majority of cases, the dose prescription requirements were within the acceptable range. In summary, the results of this study indicate that the PRIDE concept is capable of meeting its constraints and achieving adequate dose coverage. Furthermore, the increase of RN risk, estimated by the logarithmical NTCP comparison, is within the acceptable range. Therefore, if the risk of RN through BEV by the same factor as it increased by dose escalation holds true, isotoxicity could be achievable in the PRIDE trial.

## 5 Conclusion

The concept of the PRIDE trial holds promise for a significant improvement in the recurrence rate and OS in glioblastoma patients. By escalating the radiation dose to the morphologically and biologically defined tumor volume, while reducing margins and implementing the protective application of BEV, the PRIDE trial aims to enhance treatment outcomes without increasing treatment-related toxicity. The dosimetric feasibility, demonstrated in the presented data, even for critical tumor locations, is encouraging. It remains to be seen whether the PRIDE trial will be able to validate its hypothesis of achieving isotoxicity through the concomitant application of BEV, despite dose escalation.

## CRediT authorship contribution statement

**Raphael Bodensohn:** Formal analysis, Investigation, Writing – original draft, Visualization. **Daniel F. Fleischmann:** Investigation, Writing – review & editing. **Sebastian H. Maier:** Data curation, Visualization. **Vasiliki Anagnostatou:** Software, Visualization, Writing – review & editing. **Sylvia Garny:** Investigation, Writing – review & editing. **Alexander Nitschmann:** Software. **Marcel Büttner:** Visualization. **Johannes Mücke:** Writing – review & editing. **Stephan Schönecker:** Software. **Kristian Unger:** Writing – review & editing. **Elgin Hoffmann:** Writing – review & editing. **Frank Paulsen:** Writing – review & editing. **Daniela Thorwarth:** Writing – review & editing. **Adrien Holzgreve:** Resources, Writing – review & editing. **Nathalie L. Albert:** Resources, Writing – review & editing. **Stefanie Corradini:** Writing – review & editing. **Ghazaleh Tabatabai:** Writing – review & editing. **Claus Belka:** Resources, Supervision. **Maximilian Niyazi:** Conceptualization, Methodology, Validation, Writing – review & editing, Supervision, Project administration, Funding acquisition.

## Declaration of competing interest

The authors declare that they have no known competing financial interests or personal relationships that could have appeared to influence the work reported in this paper.
